# Albumen in the Urine

**Published:** 1880-08

**Authors:** 


ALBUMEN IN THE URINE.—It is a common observation that albumen, in greater or less quantities, is found in the urine of persons who apparently enjoy the very best health —a latent albuminuria, as it were.

Prof. Johnson says: In the first place, this latent albuminuria, when associated with no other functional disturbance or structural lesion, can, in a great many instances, be traced to some probable exciting cause; in the second place, that the appearance of even the slightest trace of albumen in the urine, is always pathological, never physiological; and that the disregarding of this sign of a pathological condition is often productive of pernicious results, because a temporary and occasional albuminuria may eventuate, sooner or later (sometimes not for several years) in a total degeneration. The cause of this albiminuria may often be traced to an acute nephritis (of some months' standing,) with or without dropsical symptoms, and this nephritis may have arisen in consequence of a thorough drenching or as a sequela of scarlatina, measles, diphtheria, erysipelas, typhus fever, pyæmia, etc. In such cases, careful observation may demonstrate the presence of albumen lasting for years. The frequent occurrence of albuminuria in school children is, as Johnson says, due to the fact that they are so often exposed to cold and wet. Another not uncommon cause of this affection in boys and young people generally is the habit of remaining too long in cold baths. The writer referred to is of the opinion that if the urine of all persons who take sea baths and remain long enough to become blue and cold, could be examined several hours after a bath, the greatest portion of it would be found to contain albumen. Among the common causes of albuminuria is the excessive use of animal food and alcohol. These cases are frequently latent. Alcoholic intoxication itself may produce a temporary albuminuria. In such cases appearances of disturbed digestion exist for months or years. The urine which has been high-colored for a long time, was very acid and contained large quantities of uric and oxalic acid salts, now becomes pale, abundant, with low specific gravity, and the sediment contains tube-casts. In such cases there follow, hyperæmia of the kidney, albuminuria and finally degeneration of the kidney substance. The last result comes from the long continued elimination, through the kidneys, of the products of a defective digestion, such as the elimination of sugar in diabetes. From the primary disturbances of the liver, associated with icterus there arise, secondarily through the elimination of bile disturbances of kidneys, shown in the appearance of epithelium, tube-casts and traces of albumen. Further, the excessive use of tobacco is productive, secondarily, of albuminuria. The cause is less frequent than the others, and arises from the excitation of hepatic and gastric disturbances; it sometimes tends towards an incurable degeneration of the kidneys. The albuminuria which produces mental disturbance, appears to be indirectly dependent upon dyspepsia. Johnson's experiments show that the incidental appearances of even small particles of albumen in the urine after eating, exercise or exposure to cold, almost certainly tend to a persistent albuminuria and total degeneration of the kidneys; provided, of course, this albumen cannot be traced to some exciting cause. Years must elapse before the disease of the kidneys shows itself.

The following case is remarkable: A young man had, in his 15th year, scarlatina with dropsy. Five years later, while studying medicine, he made an examination of his urine and found an abundance of albumen. The albuminuria lasted until his death, which occured in his 45th year. During the whole time he was well and engaged in a large and perplexing practice. Johnson concluded by saying that he knew a similar case which ended in complete recovery.—*Medizinische Nenigkeiten.*

